# The impact of prenatal screening tests on prenatal diagnosis in Taiwan from 2006 to 2019: a regional cohort study

**DOI:** 10.1186/s12884-021-04360-w

**Published:** 2022-01-09

**Authors:** Ching Hua Hsiao, Ching Hsuan Chen, Po Jen Cheng, Steven W. Shaw, Woei Chyn Chu, Ran Chou Chen

**Affiliations:** 1 Department of Obstetrics and Gynecology, Taipei City Hospital, Women and Children Campus, 155 Linong Street, Sec. 2, Beitou, Taipei, 112 Taiwan; 2 Departmen of Biomedical Engineering, National Yang Ming Chiao Tung University, 155 Linong Street, Sec. 2, Beitou, Taipei, 112 Taiwan; 3 Department of Obstetrics and Gynecology, Chang Gung Memorial Hospital- Linkou Medical Center, Chang Gung University College of Medicine, Taoyuan, Taiwan; 4 Department of Obstetrics and Gynecology, Chang Gung Memorial Hospital- Taipei Medical Center, Chang Gung University College of Medicine, Taoyuan, Taiwan; 5 Department of Biomedical Imaging and Radiological Sciences, National Yang Ming Chiao Tung University, Taipei, Taiwan

**Keywords:** Combined first-trimester screening (cFTS), Second-trimester serum screening, Prenatal cytogenetic diagnosis, Non-invasive prenatal test (NIPT)

## Abstract

**Background:**

The purpose of this study is to evaluate the impact of prenatal screening tests on prenatal diagnosis in Taiwan’s 14 years from 2006 to 2019.

**Methods:**

The prenatal screening methods evolved from the second-trimester serum screening to combined first-trimester screening (cFTS) and then followed by the non-invasive cell-free DNA prenatal test (NIPT). The data used by the Department of Statistics, the Ministry of Health and Welfare and Department of Household Registration, Ministry of the Interior public website.

**Results:**

This regional registry-based cohort retrospective study examined a total of 2,775,792 births from January 2006 to December 2019. The proportion of advanced maternal age (AMA) pregnancies increased from 11.63% in 2006 to 30.94% in 2019. Overall, invasive diagnostic testing was used in 87.22% of AMA pregnancies. The prenatal detection rate of trisomy 21 and 18 increased from 74.1% and 83.3% in 2006 to 96.9% and 98.8% in 2019, respectively.

**Conclusion:**

During the second-trimester and cFTS periods, the percentage of AMA pregnancies increased every year and the number of invasive procedures also accompany with increased percentage of AMA. However, during the period that NIPT were implemented, the percentage of invasive procedures decreased.

## Introduction

Down Syndrome (DS) is one of the most common causes of mental retardation and congenital abnormalities. In 1988, Wald et al. introduced triple screening, which examines maternal serum alpha-fetoprotein (MSAFP), estriol (E3), and human chorionic gonadotropin (hCG). Triple screening builds on the maternal age-specific a priori risk for DS and refines it using three serum markers [1]. Double (AFP and hCG) and triple (plus uE3) serum screening have been implemented in Taiwan since 1994 and have been liberally used thereafter [2]. Jou et al. reported that the detection rate is about 60% in Taiwan. The ratio of DS live births to total cases of DS subsequently dropped significantly from 70.42% in 1994 to 48.74% in 2001 [3].

The combined first-trimester screening (cFTS) for trisomy 21 uses a combination of maternal age, maternal serum free β-human chorionic gonadotropin (β-hCG) and pregnancy-associated plasma protein-A (PAPP-A) concentration between 8 and 14 weeks, and fetal nuchal translucency (NT) at 11 ^+0^ to 13 ^+6^ weeks of gestation. This combination method provides a detection rate of around 90% for trisomy 21 with a false-positive rate (FPR) of 5% [4, 5]. In addition, a combination with ultrasound markers, such as nasal bone, tricuspid, and ductus venosus flow provide the most economic approach with increased rates of trisomy 21 detection and decreased false-positive rates [5]. The nuchal translucency training course of cFTS in prenatal screening started in 2006, while second-trimester quadruple screening started in 2008, and both types of screening have been widely used since 2010 in Taiwan [5, 6]. Shaw et al. reported on the usage of screening methods ranging from double screening to non-invasive prenatal testing based on 20 years of experience in Taiwan. Their findings indicated that the usage has increased year by year [7].

Since the feasibility of non-invasive prenatal test (NIPT) was first demonstrated, it has transitioned from research setting to clinical care. The clinical adoption of NIPT for chromosomal aneuploidy screening has already had a global effect, which has been driven by the desires of pregnant women for safer prenatal screening, as well as commercial incentives [8]. .The NIPT has been used in the first-stage test at 10–14 weeks of gestation in Taiwan since 2013 [9].

The emergence of amniocentesis and amniotic cell culture for fetal chromosomal and metabolic disorders in 1966 changed the practice forever. The application of relevant prenatal diagnosis methods has been increasing along with the cases of advanced maternal age (AMA), and the AMA indication for cytogenetic prenatal diagnosis has been a risk factor for fetal chromosome abnormalities in most countries in the past 30 years. The public health policy in Taiwan has also taken this into account [3].

This study aims to assess the impact of prenatal screening tests on invasive procedures in Taiwan from 2006 to 2019 and to survey the effects of prenatal screening tests on trisomy 21, 18, and 13 detection rates.

## Methods

The indications for prenatal cytogenetic diagnosis were consistent from 2006 to 2019 and included (A) 35 years and older at the expected time of delivery (AMA); (B) trisomy 21, 18, or 13 in the first or second-trimester screenings indicated by results of 1:270 or higher; (C) ultrasound scan anomalies with suspected chromosomal abnormalities, including increased nuchal translucency over 3.5 mm and absent nasal bone; (D) elevated MS-AFP; (E) parents with chromosomal balanced rearrangements; (F) a previous child with chromosomal or congenital abnormality; (G) family history of DNA abnormality or metabolic disorder; and (H) extreme anxiety of the pregnant woman. Informed consent was obtained from every pregnant woman before performing invasive prenatal cytological diagnosis at an individual diagnostic center or hospital. The costs for cytogenetic prenatal diagnosis are partly (62.5%) covered by national health insurance. The number of invasive prenatal diagnosis procedures and the outcomes record of amniocentesis, CVS, or cordocentesis were sent to the Taiwan Central Cytogenetic Registry of the Health Promotion Administration register center. Live births and Terminations of Pregnancies (ToPs) among infants with trisomy 21, trisomy 18, and trisomy 13 were recorded for this period by the Taiwanese National Birth Defect Registration and Notification system. The data used in this study from both of the Department of Household Registration, Minister of the Interior [10] and Department of Statistics, Ministry of Health and Welfare System [11] and all data were fully anonymized. This study was conducted by directly analyzing the information documented in this database. The study was approved by the research ethics committee of Taipei City Hospital (TCHIRB-11012009-W).

The groups difference was evaluated using Chi-square test and pairwise comparison were conducted using proportional test with Bonferroni correction. Relationship between the invasive procedure rate and trisomy was conducted with Pearson correlation. A test was considered statistically significant if a *p*-value was <0.05.

## Results

There were 2,775,792 live births from 2006 to 2019 in Taiwan, and invasive prenatal diagnosis procedures were performed in 618,846 cases (22.28% of all cases). The proportion of pregnant women with AMA ≧ 35 years increased from 11.63% (23,920/205,720) in 2006 to 30.94% (54,162/175,074) in 2019 are summarized in Table 1. Among AMA pregnancies, the invasive procedure rate was about 87.22% (498,863/618,846) (66.0% ~ 99.6%, *p* < 0.01) in this period. In pregnant women less than 35-years-old, the invasive procedure rate revealed no significant change with an average rate of 5.7% (4.4% ~ 6.9%, *p* = 0.15).

**Table 1:** Total registered live births and prenatal diagnostic tests for AMA pregnancies from 2006 to 2019

	2006	2007	2008	2009	2010	2011	2012	2013	2014	2015	2016	2017	2018	2019	Total/Average
Registered live births	205,720	203,711	196,486	192,133	166,473	198,348	234,599	194,939	211,399	213,093	207,600	194,616	181,601	175,074	2,775,792
Maternal age ≧ 35	23,920	25,260	26,389	27,683	28,689	39,213	48,653	42,787	48,689	53,076	56,207	56,508	54,406	54,162	585,642
% of maternal age ≧ 35	11.63%	12.40%	13.43%	14.41%	17.23%	19.76%	20.74%	21.95%	23.03%	24.91%	27.07%	29.04%	29.96%	30.94%	21.18%
Total No. invasive prenatal diagnosis	30,302	32,801	33,396	38,632	37,542	48,317	56,117	47,117	51,421	57,471	53,919	46,413	41,520	43,878	618,846
% of total invasive prenatal diagnosis	14.73%	16.10%	17.00%	20.11%	22.55%	24.36%	23.92%	24.17%	24.32%	26.97%	25.97%	23.85%	22.86%	25.06%	22.28%
No. of maternal age≧ 35	21,278	23,628	23,991	27,571	28,024	38,127	45,318	38,921	43,017	48,547	45,929	40,124	35,932	38,456	498,863
% of invasive maternal age≧ 35	88.95%	93.54%	90.91%	99.60%	97.68%	97.23%	93.15%	90.96%	88.35%	91.47%	81.17%	71.00%	66.04%	71.00%	87.22%
Total Trisomy 21	228	182	219	211	211	322	312	321	359	366	361	345	327	333	4097
Registered trisomy 21 TOPs	169	132	173	163	191	291	288	289	334	343	346	331	317	321	3688
Registered trisomy 21 live birth	59	50	46	48	20	31	24	32	25	23	15	14	10	12	409
Total Trisomy 18	42	31	29	32	70	97	97	83	98	112	108	97	102	81	1079
Registered trisomy 18 TOPs	35	29	26	27	68	92	94	82	97	111	107	95	100	80	1043
Registered trisomy 18 live births	7	2	3	5	2	5	3	1	1	1	1	2	2	1	36
Total trisomy 13	9	8	13	7	25	21	19	36	30	33	29	32	28	27	317
Registered trisomy 13 TOPs	9	3	13	6	23	20	19	36	28	32	29	32	28	27	305
Registered trisomy 13 live births	0	5	0	1	2	1	0	0	2	1	0	0	0	0	12

The prenatal rate of trisomy 21 births avoided increased from 74.1% (169/228) in 2006 to 96.4% (321/333) in 2019. The total number of trisomy 21 cases was 4,097 and the population birth prevalence increased from 11.1/10,000 in 2006 to 19.0/10,000 in 2019. The total number of other chromosomal abnormalities was 1079 cases for trisomy 18 and 317 cases for trisomy 13. Both abnormalities showed increasing population birth prevalence from 2006 to 2019 (2.0/10,000 to 4.6/10000 in trisomy 18, 0.4/10,000 to 1.5/10000 in trisomy 13). The rates of trisomy, 18 and 13 births avoided were 83.3% (35/42) and 100% (9/9) in 2006, and 98.8% (80/81) and 100% (27/27) in 2019, respectively shown in Table.

**Fig. 1:**
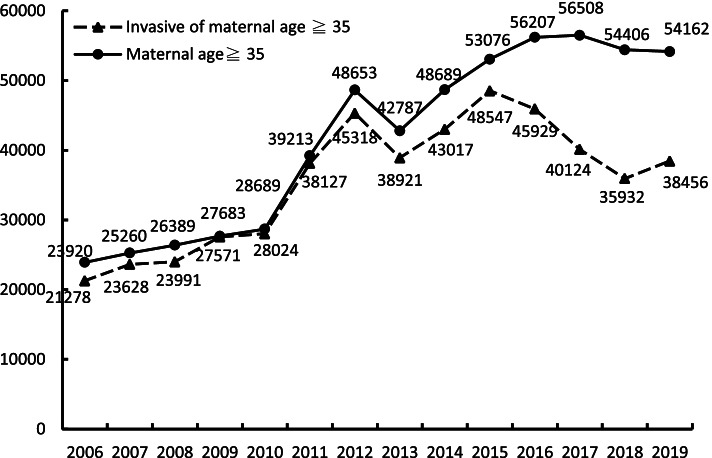
Numbers of advanced maternal age (≧35) pregnancies and invasive diagnostic tests performed between 2006 and 2019

The relationship between the invasive procedure rate and chromosomal abnormality births avoided was assessed separately for (A) 2006 to 2010, (B) 2011 to 2015 and (C) 2016 to 2019 three groups overall trend to Table 2. The number of total invasive prenatal diagnoses revealed a trend towards an increase in the double/triple and cFTS/quadruple test from 2006 to 2015 and a trend towards a decrease in the duration of implement NIPT from 2016 to 2019. In 2016-2019, the number of AMA pregnancies substantially increased. However, along with the decrease in invasive procedures, these changes lead to a sudden drop in related NIPT screening shown in Fig. 1. (Chi-Square Test *p* < 0.001, Pairwise Comparison B > C > A).

**Table 2:** Comparison of data for Double/Triple, cFTS/Quadruple and NIPT screening tests

Grouped period screening policy	A. 2006-2010 Double/Triple	B. 2011-2015 cFTS/quadruple	C. 2016-2019 NIPT	Chi-Square Test *p* value	Pairwise Comparison (Proportional test with Bonferroni correction)
Registered live births	964523	1052378	758891		
Maternal age≧ 35 (%)	131941 (13.7)	232418 (22.1)	221283 (29.2)	<0.001	C > B > A
Total No. invasive prenatal diagnosis	172673	260443	185730		
Maternal age≧ 35 (%)	124492 (72.1)	213930 (82.1)	160441 (86.4)	<0.001	C > B > A
Invasive diagnosis rate					
All age	17.90%	24.75%	24.47%	<0.001	B > C > A
Maternal age≧ 35	94.35%	92.05%	72.50%	<0.001	A > B > C
Total Trisomy 21	1051	1680	1366		
Trisomy 21 livebirths (%)	223 (21.2)	135 (8.0)	51 (3.7)	<0.001	A > B > C
Total Trisomy 18	204	487	388		
Trisomy 18 livebirths (%)	19 (9.3)	11 (2.3)	6 (1.5)	<0.001	A > (B , C)
Total trisomy 13	62	139	116		
Trisomy 13 livebirths (%)	8 (12.9)	4 (2.9)	0	<0.001	A > (B , C)

The across three major time periods tests use the Pairwise Comparison (Proportional test with Bonferroni correction)

**Fig. 2:**
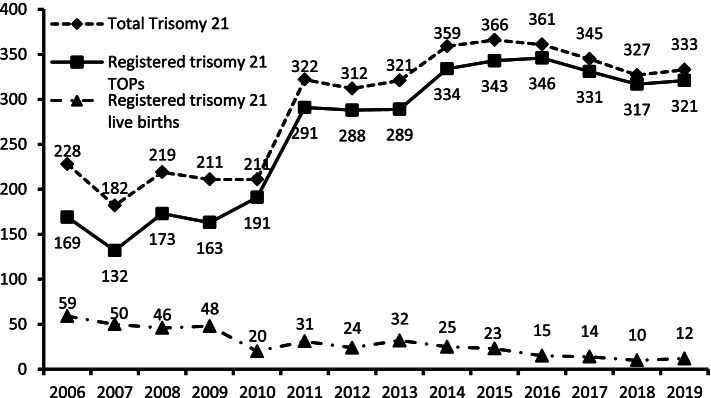
The amount of total trisomy 21, registered termination of pregnancies (TOPs) and live births from 2006 to 2019

Until the end of 2015, the invasive procedure rate was positively correlated with the rates of trisomy 21, trisomy 18, and trisomy 13 births avoided. After 2015, a negative correlation was observed between the invasive procedure rate with all three trisomies (*r* = -0.96, -0.37, -0.73 for trisomy 21, 18, 13, all *p* < 0.001). Combined with the high ToPs and decreased trisomy 21 live birth rates overall trend to Fig. 2, these findings suggest an efficiency from 2016 – 2019.

## Discussion

Hospitals offer different types of screening tests for DS for different gestational ages, which are selected by pregnant women after counseling. In prenatal screening, the use of the double/triple, cFTS, quadruple and NIPT screening tests gradually changed over the period from 2006 to 2019. During 2006 to 2010, the double screening (AFP, HCG) or triple screening (AFP, HCG, uE3) were performed in the second trimester [4]. cFTS is the most commonly offered test in the first trimester from 2011 to 2015. A serum test (PAPP-A and free beta HCG) is performed during early pregnancy at gestational weeks 9–14. Nuchal translucency (NT) is measured, and general ultrasound screening is performed at gestational weeks 11^+0^–13^+6^ [5, 6]. NIPT tests were offered in the first trimester from 2015 onwards. While the costs of screening are not reimbursed by national health insurance, the screening is commonly accepted by pregnant women.

The cFTS and quadruple screening protocols have been popular since in 2010, after which the total amount of prenatal screening and detection rates increased in prenatal examinations. Not only are an extended number of sonographic markers being used in cFTS but also the NIPT in the first trimester, which has greatly increased the sensitivity and decreased the FPR for the detection of chromosomal abnormalities [6, 7, 9, 10]. .As shown in this study, the percentage of live births among total trisomy 21 cases decreased from 25.88% (59/228) in 2006 to 3.60% (12/333) in 2019 are summarized in Table 1.

In 1978, the Conference on Amniocentesis of the National Institutes of Health Consensus Development Program recommended that screening be offered to pregnant women aged 35 years or over in the United States [12]. The indications for prenatal cytogenetic diagnosis were announced thereafter by the Bureau of Health Promotion Administration of the Ministry of Health and Welfare of Taiwan. DS births among AMA women were significantly higher than women of the average maternal age for childbirth among the population [12, 13]. In Taiwan, pregnant women aged 35 or more represented 4.8% of all pregnant women in 1993 and this rate increased steadily to 8.3% by 2001 [4]. However, the population of AMA increased from 11.63% (23,920/205,720) in 2006 to 30.94% (54,162/175,074) by 2019 (see Table 1).

In Taiwan, the major indication for invasive procedures is AMA with a rate of 57.29% during the 1995 to 2004 period [14]. In this study, the percentage of invasive procedures increased every year alongside the increase in AMA. The official policy indications recommend invasive amniocentesis or CVS cytogenetic diagnosis in cases of AMA, and the cost is partly reimbursed (62.5%) by national health insurance. Many pregnant women with AMA are referred for cytogenetic diagnosis. In the Central Cytogenetic Registry database, the total number of prenatal diagnosis cases was 618,846 during the 2006 to 2019 study period. Acceptance of the procedures among pregnant women increased from 14.73% in 2006 to 26.97% in 2015, and among AMA pregnancies, the invasive procedure rate was about 92.88% in this period. This data indicates that positive progress in the invasive procedure rate along with AMA result in Taiwan official strategy recommend the AMA direct for amniocenteses and regardless Double, Triple, cFTS and quadruple test. However, after NIPT became more widely used, the invasive procedure rate among AMA pregnancies decreased each year from 81.71% in 2016, to 71.00% in 2017, to 66.04% in 2018 and finally to 71.00% in 2019 are summarized in Table 1.

The estimated second-trimester prevalence of trisomy 21 was 1 in 740 as of 1974, but by 1997, it had increased to 1 in 504 [15–17]. According to Jou et al., two- or three-marker maternal serum screening tests have been liberally used for trisomy 21 during the second trimester since 1993. This sudden fall in live birth rates of infants with trisomy 21 corresponds to a decrease of nearly 60.0% in the birth prevalence in Taiwan. A marked decrease in the live birth rates of cases with trisomy 21 occurred between 1994 and 2001 from 0.63 per 1000 births to 0.23 per 1000 births. In 1993, 76.9% of cases diagnosed with trisomy 21 were born alive, in contrast to 32.5% in 2001 [4]. According to the data in this study, first-trimester prenatal screening has been used since 2010, and ToPs in cases of trisomy 21, 18, and 13 have increased, while live births have decreased dramatically (see Table 1). The total numbers of ToPs in cases of trisomy 21, 18, and 13 were 169, 35, and 9 cases in 2006 and 321, 80, and 27 cases in 2019, respectively. The numbers of live births in cases of trisomy 21, 18, and 13 were 59, 7, and 0 cases in 2006 and 12, 1, and 0 cases in 2019, respectively. The population incidence (1/10,000) increased in cases of trisomy 21, 18, and 13 from 11.08%, 2.04%, and 0.44% in 2006 to 19.02%, 4.63%, and 1.54% in 2019, respectively. The incidence is based on live births and ToPs in cases of trisomy 21, 18, and 13 and without including the miscarriage loss of registration report. This resulted from the greater rates of ToPs at an early stage and early diagnosis and the attitude of most parents to avoid trisomy 21 births.

The use of NIPT is widely spreading in prenatal screening and it is highly accurate in detecting fetal trisomy 21, 18, and 13 with both sensitivity and specificity >99% [9, 18–20]. It can also detect fetal CNVs > 5 Mb with high sensitivity, provided that the fetal fraction is high enough, without increasing the sequencing depth. The detection power of NIPT is mostly determined by fetal fraction and CNV size [21]. However, Norton et al. reported on 452,901 women who underwent sequential screening comparisons of NIPT screening regardless of “no result” assumed normal or high risk, that sequential test (81.6%) with detection rate greater than NIPT (77.1%) screening [22]. Therefore, the NIPT results with or without a “no result” report for aneuploids or CNV screening must be interpreted with caution and validated by additional diagnostic studies. NIPT screening was first implemented in 2013 in Taiwan and has shown good outcomes in a preliminary report by Shaw et al. [9]

As of 2016, the American College of Obstetrics and Gynecology had recommended that all pregnant women be offered prenatal screening for Down syndrome, regardless of age [23]. Egan et al. suggest that the practice of offering routine amniocentesis to women of age 35 years or older without first performing maternal serum screening is outdated and should be abandoned [15]. The marked decrease in DS live births in Taiwan from 1993 to 2010 is the result of the policies of the prenatal diagnosis program, which includes amniocentesis for pregnant women aged 35 or more and the liberal application of maternal serum screening for DS in younger women [4, 7]. No adjustments to this policy have been implemented during the study period.

In a systematic review and meta-analysis by Akolekar et al., the miscarriage rate was about 0.11-0.22% rate in cases of invasive procedures including amniocentesis and CVS [24]. The data indicate that some cases of miscarriage unfortunately occur every year due to the procedures. Due to cultural differences in Taiwan, the majority of families have negative attitudes towards children with chromosomal abnormalities and prefer to avoid DS births, resulting in high rates of invasive procedures. Not to mention, the substantial increase of AMA cases further increases the number of amniocentesis procedures performed. NIPT, on the other hand, has three primary advantages with the highest detection rate (99%), capable of being performed at any gestational age after 9-10 weeks, and results in the lowest false positive rate [25]. In a Swedish Cohort study, it was revealed that positive attitudes were present towards NIPT, which is now encouraged in prenatal screening [25, 26]. With a low false positive rate, NIPT consequently decreases the potential collateral damage on procedure-related losses and costs. Combined with the high detection rate, pregnant women have become more accepting towards NIPT. Therefore, the acceptance of invasive diagnostic procedures by AMA women decreased from 91.47% to 70.00% in 2015 to 2019 in Taiwan. The high efficacy NIPT result in women attitude change and decreased by invasive procedures to avoid miscarriages of the collateral damage.

## Conclusion

The outdated policy practice of offering routine prenatal diagnosis, invasive procedures have increased alongside the number of AMA women in Taiwan from 2006 to 2015. That are results in (1) Taiwan official strategy recommend the AMA direct for amniocenteses and regardless Double, Triple, and cFTS test, (2) the fee for cytogenetic prenatal diagnosis is partially reimbursed (62.5%) by national health insurance, and (3) most parents prefer to avoid Down syndrome births. However, with the implementation of NIPT, invasive procedures began to decrease despite the continual increase of AMA pregnancies from 2016 to 2019. It is result in the attitude of invasive procedures collateral miscarriages avoid. This was also accompanied by an increase of prenatal diagnosis as a result of higher trisomy 21, 18, and 13 detection rates.

## Data Availability

The data are not publicly available due to restrictions as they contain information that could compromise the privacy of research participants but are available encoded from the corresponding author on reasonable request.

## Abbreviations

cFTS: Combined first-trimester screening
NT: Nuchal translucency
AMA: Advanced maternal age
CNV: Copy number various
NIPT: Non-invasive prenatal test
ToP: Termination of Pregnancy
